# Solvent-free enzymatic synthesis of 1, 3-Diacylglycerols by direct esterification of glycerol with saturated fatty acids

**DOI:** 10.1186/1476-511X-12-65

**Published:** 2013-05-08

**Authors:** Nanjing Zhong, Zhongyu Gui, Li Xu, Jianrong Huang, Kun Hu, Yongqing Gao, Xia Zhang, Zhenbo Xu, Jianyu Su, Bing Li

**Affiliations:** 1 School of Food Science, Guangdong Pharmaceutical University, Zhongshan, China; 2 College of Light Industry and Food Science, South China University of Technology, Guangzhou, China; 3 School of Chemistry and Chemical Engineering, Guangdong Pharmaceutical University, Zhongshan, China; 4 Department of Microbial Pathogenesis, Dental School, University of Maryland-Baltimore, Baltimore, MD, 21201, USA

**Keywords:** 1, 3-diacylglycerol, Air-bubbling, Enzyme, Synthesis

## Abstract

**Background:**

Pure 1, 3-diacylglycerols (1, 3-DAG) have been considered to be significant surfactants in food, cosmetics and pharmaceutical industries, as well as the effect on obesity prevention.

**Methods:**

In this study, a vacuum-driven air bubbling operation mode was developed and evaluated for the enzymatic synthesis of 1, 3-DAG of saturated fatty acids, by direct esterification of glycerol with fatty acids in a solvent-free system. The employed vacuum-driven air bubbling operation mode was comparable to vacuum-driven N_2_ bubbling protocol, in terms of lauric acid conversion and 1, 3-dilaurin content.

**Results:**

Some operation parameters were optimized, and 95.3% of lauric acid conversion and 80.3% of 1, 3-dilaurin content was obtained after 3-h reaction at 50°C, with 5 wt% of Lipozyme RM IM (based on reactants) amount. Of the lipases studied, both Lipozyme RM IM and Novozym 435 exhibited good performance in terms of lauric acid conversion. Lipozyme TL IM, however, showed low activity. Lipozyme RM IM showed good operational stability in this operation protocol, 80.2% of the original catalytic activity remained after 10 consecutive batch applications. Some other 1, 3-DAG were prepared and high content was obtained after purification: 98.5% for 1, 3-dicaprylin, 99.2% for 1, 3-dicaprin, 99.1% for 1, 3-dilaurin, 99.5 for 1, 3-dipalmitin and 99.4% for 1, 3-disterin.

**Conclusion:**

The established vacuum-driven air bubbling operation protocol had been demonstrated to be a simple-operating, cost-effective, application practical and efficient methodology for 1, 3-DAG preparation.

## Backgrounds

With attractive intermediates for synthetic application, pure 1, 3-diacylglycerols (1, 3-DAG) have been considered to be significant surfactants in food, cosmetics and pharmaceutical industries [1,2], as well as the effect on obesity prevention. 1, 3-DAG may potentially function on building blocks for synthesis of lipid derivatives, such as phospholipids, glycolipids or lipoproteins, which have been shown to improve bioavailability and reduce side effects, so as to used as starting materials for the preparation of some drugs [3-5].

Despite its key role in industries, however, production of high yield 1, 3-DAG by chemical methods proves difficult and multi-step reaction sequences and tedious purification steps are still required, which remains the major obstacle for the broad application of 1, 3-DAG [6]. As a promising chemical methodology, enzymatic approach had been commonly employed to obtain high yield of pure 1, 3-DAG [3,7-9], and among these approaches, direct esterificaiton of glycerol with fatty acids had been widely used, where water removal remains the critical significance to shift the equilibrium toward the formation of 1, 3-DAG. Approaches to remove water generated include application of molecular sieves, nitrogen gas (N_2_) evaporation and vacuum evaporation [3,4,10,11].

**Figure 1:**
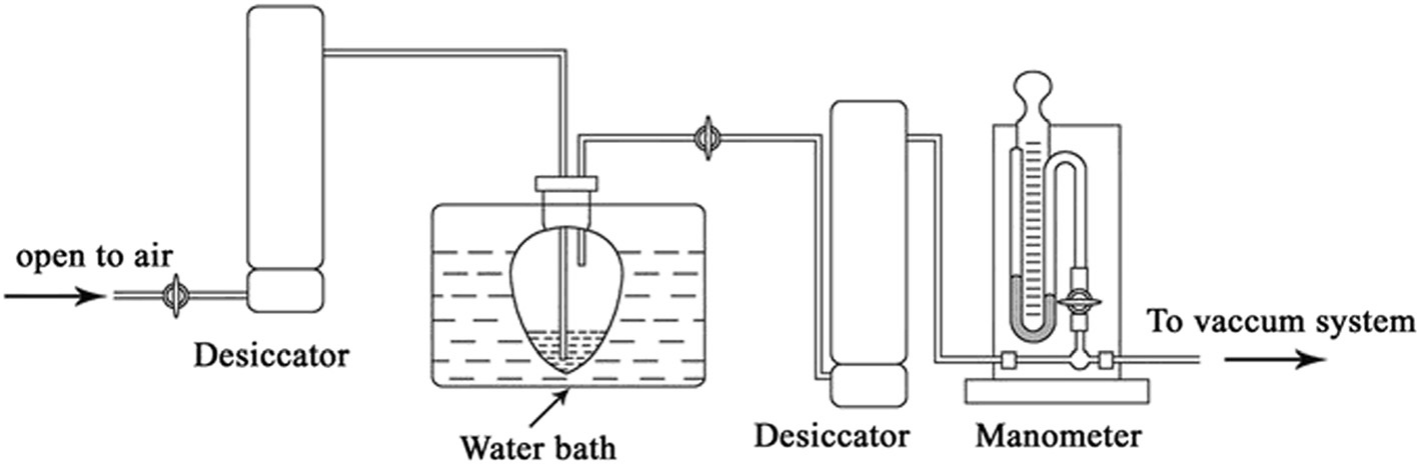
**Reaction setup.** Schematic diagram of vacuum-driven air bubbling reaction system.

Most recently, a more efficient procedure, the vacuum-driven N_2_ bubbling operation mode, for 1, 3-DAG synthesis had been developed [5,12], the procedure of which included the introduction of N_2_ to the reactor bottom, the heavy layer of hydrophilic glycerol and solid lipase “blown” up and therefore interacted with hydrophobic fatty acid for reaction acceleration, as well as the evaportated water formed during the reaction which in turn favors high conversion of fatty acid and high content of products. The protocol was advantageous over the afore-mentioned water remocal approaches, in terms of reaction rate and the content of 1, 3-DAG. Furthermore, lipase exhibited excellent stability in this protocol [5]. However, the potential danger of N_2_, expense (0.1 dollars per liter), as well as its labor demand, had restricted its further development and broad application. N_2_ especially pure N_2_ may be fatal as it leads to quick asphyxiation, which is responsible for several deaths due to nitrogen asphyxiation occur in the US every year as nitrogen is used widely. In addition, the compressed N_2_ becomes more dangerous when it comes to a fire situation, which will raise the risk of bursting, or lead to human’s unconsciousness, weakness and suffocation. Therefore, if possible, a safer, more economical and convenient operation mode was necessary for 1, 3-DAG synthesis. In this present study, the vacuum-driven N_2_ bubbling operation mode was modified for 1, 3-DAG of saturated fatty acids synthesis. To avoid the disadvantages of N_2_, the inhale tube was open to air instead of linking to compressed N_2_ (Figure 1), thus we called this modified protocol as vacuum-driven air bubbling operation mode. The protocol of which included the vacuum state of reactor formed via vacuum pump, air automatically inhaled into the reactor bottom, glycerol layer and solid lipase “blown” up to interact with fatty acid for reaction acceleration, water then formed during the reaction evaporated, and thus, high conversion of fatty acid and high content of product obtained. In the present work, the developed protocol was evaluated by comparison with N_2_ bubbling procedure, lauric acid was used as the model fatty acid, and effect of some operation parameters on the reaction had also been investigated. In addition, evaluation of lipase stability and purification of the product had been further performed.

## Methods

### Reagents

Glycerol with a purity of more than 99.0% was purchased from Guangzhou Chemical Reagent Factory (Guangzhou, China). Saturated fatty acids used in this experiment were all from Shanghai Reagent Co. Ltd with a purity of more than 99.0% (Shanghai, China). The *sn*-1, 3 specific Lipozyme RM IM (immobilized *Rhizomucor miehei* lipase), Lipozyme TL IM (immobilized *Thermomyces lanuginosus* lipase) and Novozym 435 (immobilized *Candida antarctica* B lipase) were obtained from Novozymes (Beijing, China). All other solvents and reagents were analytical or chromatographic grades.

### 1, 3-DAG synthesis

The reaction blends consisted of 10 mmol glycerol, 20 mmol fatty acid and 5 wt% of lipase based on reactants, were incubated in a 50 mL pear-shaped flask (Figure 1). Reaction temperature was controlled by water bath, with vacuum at 4 mm Hg applied throughout the reaction. Lauric acid was used as model fatty acid, and the reaction temperature was 50°C unless otherwise stated. The reaction was initiated by the application of vacuum, once the vacuum state of reactor formed via vacuum pump, air automatically inhaled into the reactor bottom, glycerol layer and solid lipase “blown” up to interact with fatty acid, thus the reaction proceeded. At approximate time intervals, 20 μL of samples were withdrawn for lipid profiles analysis.

### Determination of lipid profiles

Content of free fatty acid was determined by KOH titration according to the standard method [13,14]. The conversion of fatty acid was defined as the esterified fatty acid amount to the initial used fatty acid amount. Meanwhile, lipid profile was analyzed by a normal-phase high-performance liquid chromatography (NP-HPLC). The chromatography apparatus equipped with a binary waters 515 HPLC pump and a Waters 2410 differential refractive index detector. The separation of the compounds was performed on a phenomenex normal phase luna silica column (250 × 4.6 mm i.d., particle size 5 μm) and the column temperature was hold constant at 35°C. The mobile phase was *n*-hexane–2-propanol (15:1) at flow rate of 1.0 mL/min. Samples were dissolved in mobile phase (5 mg/mL) and 20 μL aliquots were injected for HPLC analysis with double determinations.

### Reusability of lipase

The reusability of lipase under the present protocol was studied. The model reaction had been performed with 20 mmol lauric acid, 10 mmol glycerol, and 0.25 g Lipozyme RM IM at 50°C, with vacuum at 4 mm Hg. The reaction was progressed for 3 h for each cycle. At the end of the reaction, lipase was isolated by filtration and then used for the next batch under otherwise identical conditions. The relative activity of lipase was defined as the ratio of 1, 3-dilaurin content obtained from each cycle to the 1, 3-dilaurin content obtained from the fist cycle.

$$\begin{array}{l} \mathrm{Relativeactivity}\;\left(\%\right)\\ \;=\frac{\mathrm{content}\;\mathrm{of}\;1,3-\mathrm{dilaurin}\;\mathrm{obtained}\;\mathrm{from}\;\mathrm{each}\;\mathrm{cycle}}{\mathrm{content}\;\mathrm{of}\;1,3-\mathrm{dilaurin}\;\mathrm{obtained}\;\mathrm{from}\;\mathrm{the}\;\mathrm{first}\;\mathrm{cycle}}\\ \;\times 100\% \end{array}$$

### Purification of products

After reaction, the mixtures were filtrated to remove the lipase (for solid products, petroleum ether was added to the mixtures to help the filtration and then evaporated the petroleum ether), solid 1, 3-DAG was purified by recrystallization from dry methanol and liquid 1, 3-DAG purification was achieved by a short column of silica gel. The liquid reaction mixture was dissolved in a mixture of *n*-hexane and diethyl ether (1:1, v/v) and then filtered over the column [3].

### Statistical analysis

An analysis of variance (ANOVA) was performed using the SPSS 13.0 statistical analysis system, significance of differences was defined at *P* < 0.05 with Tukey’s test.

## Results

### Esterification time courses under air-bubbling and N_2_-bubbling protocols

**Figure 2:**
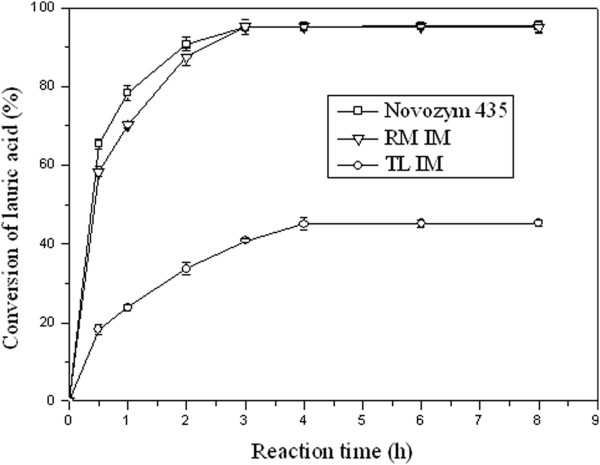
**Time courses of lauric acid conversion and 1, 3–dilaurin content under air-bubbling and N**_**2**_**-bubbling reaction systems.** The reaction was performed with 20 mmol lauric acid, 10 mmol glycerol, and 0.25 g Lipozyme RM IM at 50°C. Vacuum applied was at 4 mm Hg for both the systems and N_2_ was at 0.7 L/min for N_2_-bubbling reaction system.

The developed vacuum driven air-bubbling operation mode was evaluated by comparison with the N_2_-bubbling protocol, which was recognized as an efficient procedure for 1, 3-DAG synthesis. Esterification time courses of lauric acid under these two procedures were presented in Figure 2. The developed air-bubbling operation mode had been demonstrated to be comparable to N_2_-bubbling design, and no much difference was observed between these procedures, in terms of lauric acid conversion and 1, 3-dilaurin content. A slight decrease of 1, 3-dilaurin content during the reaction period of 4-8 h was attributed to the acylmigration. Therefore, air-bubbling design could also be considered for the preparation of 1, 3-DAG of saturated fatty acids.

### Effect of lipase on the conversion of lauric acid

**Figure 3:**
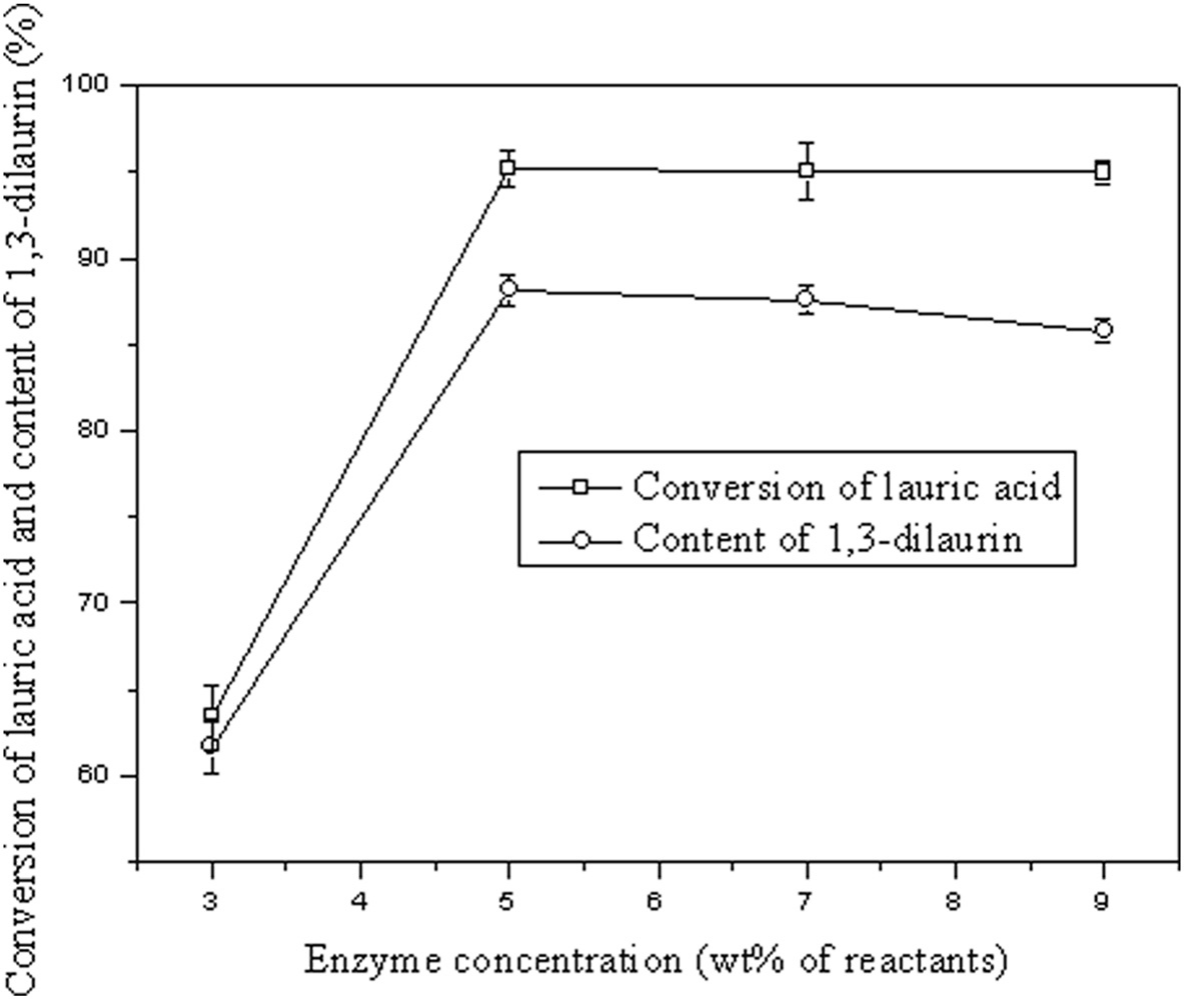
**Effect of lipase on conversion of lauric acid.** Reaction conditions: lauric acid 20 mmol, glycerol 10 mmol, lipase amount 0.25 g, vacuum applied 4 mm Hg, reaction temperature 50°C.

Three commonly commercial immobilized lipases, namely Lipozyme RM IM, Lipozyme TL IM and Novozym 435 were employed in the present study, and their effect on the conversion of lauric acid had also been illustrated (Figure 3). According to the results, highest initial reaction rate was observed with Novozym 435 as catalyst, which was attributed to none of water added to the reaction system. Unlike most lipases require certain content of water to remain high catalytic activity, Novozym 435 was able to keep high activity in dry state without water addition [15]. It was noteworthy a certain amount of water remained in Lipozyme RM IM and TL IM, which thus were able to catalyze the reaction to proceed without water addition, though with a lower initial reaction rate compared with Novozym 435. As the reaction progressed, water would be generated.

In terms of lauric acid conversion, no significant difference was observed between Novozym 435 and RM IM after 3-h reaction, suggesting that Novozym 435 and RM IM may possess similar specificity towards the lauric acid, which had also been supported by a previous study [3]. However, for Lipozyme TL IM, dramatically lower conversion of lauric acid was obtained. To further test the activity of TL IM in esterification reaction, oleic acid was employed (in the case of unsaturated fatty acids, other than air bubbling, N_2_ bubbling was adopted), while conversion of oleic acid turned out low (35.2 ± 0.8%), indicating that Lipozyme TL IM was unlikely suitable for esterification reaction, which was agreed with some previous reports demonstrating Lipozyme TL IM to be less active in esterification reaction [12,16-18]. As consequence, Lipozyme RM IM was selected for subsequent experiments.

### Effect of enzyme concentration on the conversion of lauric acid and content of 1, 3-dilaurin

**Figure 4:**
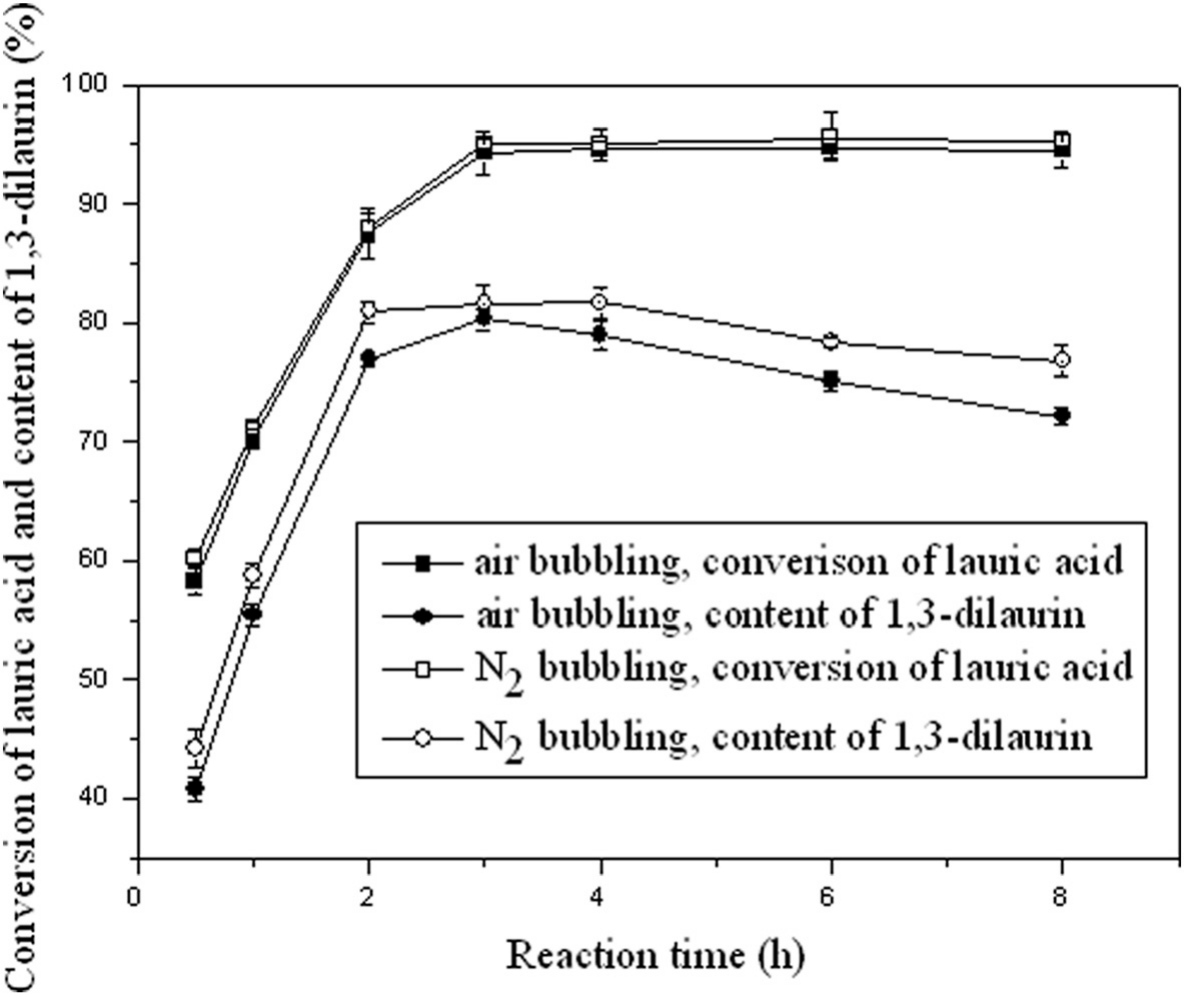
**Effect of lipase concentration on conversion of lauric acid and content of 1, 3-dilaurin.** Reaction conditions: lauric acid 20 mmol, glycerol 10 mmol, vacuum applied 4 mm Hg, reaction temperature 50°C and time 3 h.

Enzyme concentration plays a key role on the reaction rate. At certain ranges, increase in enzyme concentration leads to increase of reaction rate. In the present study, conversion of lauric acid was on the rise with Lipozyme RM IM concentration increasing from 3 to 5 wt% (based on reactants). Nevertheless, further increase enzyme amount did not lead to higher conversion of lauric acid (Figure 4), and this may be attributed to the protein aggregation of the enzyme molecules which in turn leads to the active site not expose to the substrates [19]. None of increase of lauric acid conversion may also be explained by the influence of enzyme concentration on the reaction rate. With ≥5 wt% of lipase amount, the reaction was fast and reached equilibrium with 3-h reaction; which was not observed when lipase amount lower than 5 wt%.

Content of 1, 3-dilaurin was raised with enzyme concentration increasing from 3 to 5 wt%. However, with further increasing the enzyme concentration, a slight decrease of 1, 3-dilaurin content was observed. The decline of 1, 3-dilaurin content was ascribed to the acylmigration which led to an increase of 1, 2-dilaurin content accordingly (data not shown in detail), and this was supported by previous report [5].

### Reusability of lipase

**Figure 5:**
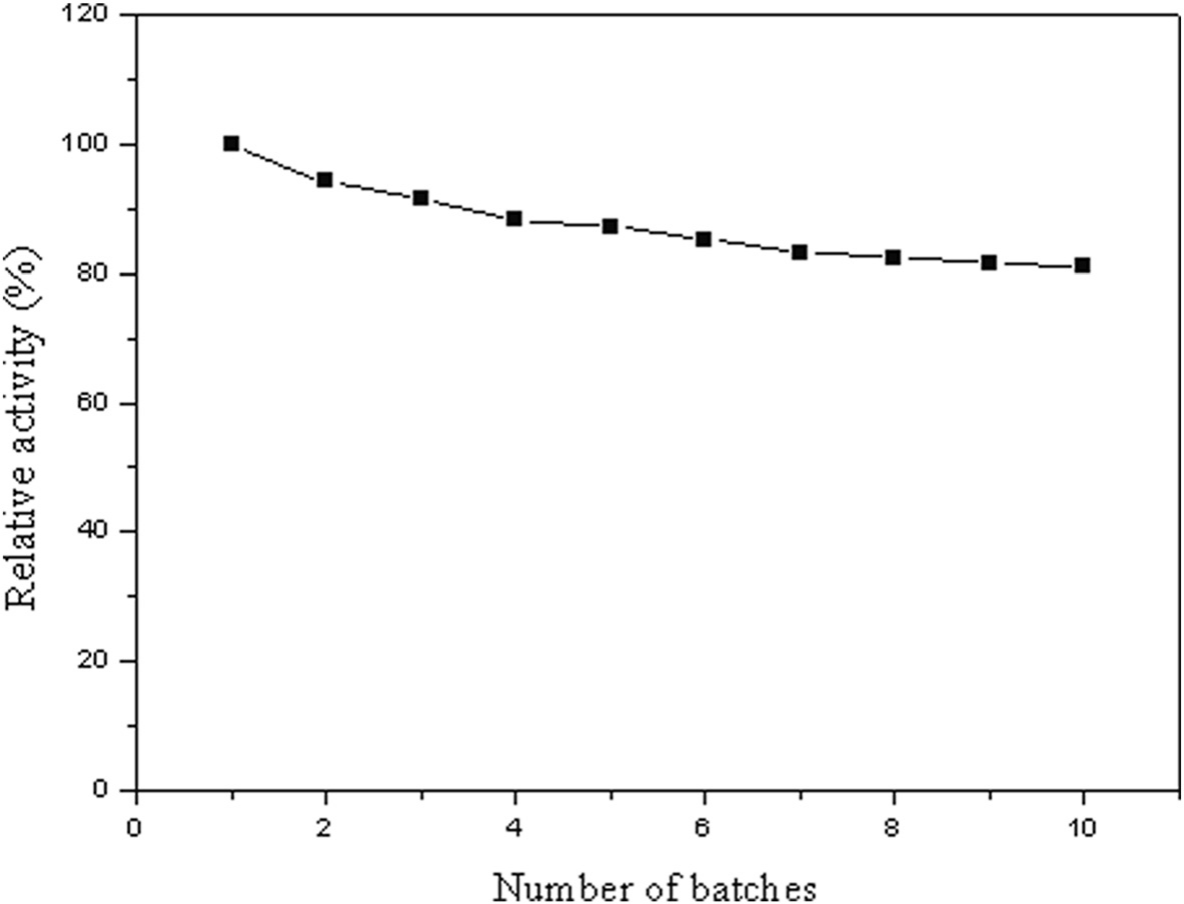
**Reusability of Lipozyme RM IM.** Reaction conditions: lauric acid 20 mmol, glycerol 10 mmol, lipase amount 0.25 g, vacuum applied 4 mm Hg, reaction temperature 50°C and time 3 h.

The reusability of the employed biocatalyst plays a critical role in practical applications. In the current study, 80.2% of the original catalytic activity remained after 10 consecutive batch applications (Figure 5). Strikingly, Guo *et al.* had preliminarily showed little loss of enzyme activity was observed after 10 batch reactions and Novozym 435 was used under the N_2_-bubbling operation mode, which may be ascribed to the more stable of Novozym 435 and/or the inert atmosphere N_2_ provided [5].

### Synthesis of 1, 3-DAG under different reaction conditions

Via the established air-bubbling mode, 1, 3-DAG of saturated fatty acids had been synthesized, with reaction conditions varied with different fatty acids. Shown from the results (Table 1), formation of liquid 1, 3-DAG was relatively low, which may be explained by either the lower activity of Lipozyme RM IM towards to the shorter-chain fatty acids or the lower temperature adopted for the shorter-chain fatty acids. Purification of liquid 1, 3-DAG was also difficult and had to be carried out twice with column chromatography. Nevertheless, high content of 1, 3-DAG was obtained after purification.

**Table 1:** **Synthesis of 1, 3-DAG**^**a **^**under different reaction conditions.**

**Fatty acid species**^**b**^	**Time (h)**	**Temperature (°C)**	**Purification**	**1,3-DAG (%)content**^**c**^
C8:0	6	40	column (twice)	98.5
C10:0	5	45	column (twice)	99.2
C12:0	3	50	recrystallization	99.1
C16:0	2	65	recrystallization	99.5
C18:0	2	70	recrystallization	99.4

^a^Reaction conditions: Fatty acid 20 mmol, glycerol 10 mmol, lipase amount 5 wt% of reactants, vacuum applied 4 mm Hg.

^b^C8:0 caprylic acid; C10:0 capric acid C12:0 lauric acid; C16:0 palmitic acid; C18:0 stearic acid.

^c^1, 3-DAG content after purification.

## Discussion

In this study, a vacuum-driven air bubbling operation for 1, 3-DAG preparation had been developed and evaluated. Regarded as a significant surfactants in food, cosmetics and pharmaceutical industries, as well as its effect on obesity prevention and potential function on building blocks for synthesis of lipid derivatives, synthesis of 1, 3-DAG has been become one of the leading concerns. As resources were concerned, comparing with unsaturated fatty acids, saturated fatty acids exhibited additional advantages on its stability in air during synthesis of 1, 3-DAG.

With Lipozyme RM IM as catalysts, 95.3% of lauric acid conversion and 80.3% of 1, 3-dilaurin content was detected, with high content of 1, 3-DAG. In addition, 80.2% of the original catalytic activity of Lipozyme RM IM remained after 10 consecutive batch applications. The established vacuum-driven air bubbling operation protocol had been demonstrated to be a simple-operating, cost-effective, application practical and efficient methodology for 1, 3-DAG preparation.

## Abbreviations

1, 3-DAG: 1, 3-Diacylglycerols; N2: Nitrogen Gas (N_2_); NP-HPLC: Normal-Phase High-Performance Liquid Chromatography.

## Competing interests

The authors declare that they have no competing interests.

## Authors’ contributions

All authors participated in the study design, experiments, data analysis and data interpretation. NZ also participated in system design, data collection and manuscript writing. ZG and LX also participated in material preparation and manuscript revision. JH, KH, YG and JS also participated in data collection and manuscript revision. ZX also participated in the system design, results analysis and interpretation, as well as manuscript revision. All authors read and approved the final manuscript.
